# Genome Sequences of Three *Enterococcus faecalis* Strains (LAB1, LAB10, and LAB11) with Probiotic, Plant Growth-Promoting, and Nitrifying Properties

**DOI:** 10.3390/microorganisms14081653

**Published:** 2026-07-29

**Authors:** Muiz Oluwatosin Akinyemi, Wahauwouélé Hermann Coulibaly, Tano Marie-Ange Sakia Mian, Paul-Alexandru Popescu, Bassey Ebenso, Hary Razafindralambo

**Affiliations:** 1 Leeds Institute of Health Sciences, University of Leeds, Leeds LS2 9LN, UK; 2 Unit for Environmental Sciences and Management, North-West University, Potchefstroom 2520, South Africa; 3 Food Science and Technology Formation and Research Unit, Biotechnology and Food Microbiology Laboratory, Nangui Abrogoua University, Abidjan P.O. Box 801 02, Côte d’Ivoire; 4 Agroforestery Trainning and Research Unit, Agro-Valorization Laboratory, Jean Lorougnon Guédé University, Daloa BP-150, Côte d’Ivoire; 5 University of Agronomic Sciences and Veterinary Medicine of Bucharest, 59 Marasti Blvd., District 1, 011464 Bucharest, Romania; 6 Microbial Processes and Interactions, TERRA Teaching and Research Centre, University of Liege, UMRt 1158 BioEcoAgro, Gembloux Agro-Bio Tech, 5030 Gembloux, Belgium; 7 ProBioLab, 5004 Namur, Belgium; 8 Faculty of Agricultural and Natural Resources, An Giang University, Vietnam National University, 18 Ung Van Khiem Street, Long Xuyen, An Giang 90000, Vietnam; 9 Ingénierie et Technologie Emergentes, Université de Vakinankaratra, Campus Vatofotsy, Antsirabe 110 BP 108, Madagascar

**Keywords:** *Enterococcus faecalis*, genome announcement, ST19, probiotics, plant growth-promoting bacteria, nitrification, aquaponics, *Oreochromis niloticus*, Ivory Coast, MLST

## Abstract

Here we report the draft genome sequences of three *Enterococcus faecalis* strains, LAB1, LAB10, and LAB11, isolated from the pond water of a tilapia (*Oreochromis niloticus*) aquaculture farm at the University Nangui Abrogoua, Abidjan, Ivory Coast. These strains were previously characterised for their probiotic, plant growth-promoting (PGP), and nitrifying properties. All three strains were assigned to sequence type ST19 by multilocus sequence typing (MLST). The draft genomes of LAB1, LAB10, and LAB11 consist of 34, 35, and 34 contigs, totalling 2.94 Mb each (GC content 37.40%). Prokka annotation predicted 2872, 2873, and 2875 protein-coding sequences (CDS) for LAB1, LAB10, and LAB11, respectively. Genomic screening revealed no vancomycin resistance genes; however, *tet(M)* and *lsa(A)* resistance determinants were identified in all three strains, located on a repUS43-type plasmid replicon. Fourteen virulence factor homologs conserved in the *E. faecalis* reference strain V583 were detected, including *Ebp* pili, gelatinase (*gelE*), *Fsr* quorum-sensing system, and capsule biosynthesis genes, but no cytolysin operon was identified. Genes associated with stress tolerance (*katA*, *sodA*), bile salt hydrolysis (*cbh*), siderophore transport (*fepC*, *fhuD*), and ethanolamine nitrogen metabolism (*eutB/eutC*) were identified in all three genomes. Pan-genome analysis with the *E. faecalis* reference strain revealed 551 core gene clusters and 279 gene clusters exclusive to the three aquaculture isolates. Despite their high genomic similarity, we report the three genomes as distinct isolates due to observed differences in their expressed phenotypic properties. These sequences provide a genomic resource supporting the development of multifunctional probiotic consortia for integrated aquaponic systems.

## 1. Introduction

Aquaponics integrates recirculating aquaculture with soilless plant cultivation in a single closed-loop system, offering a sustainable approach to food production that reduces water consumption and eliminates the need for synthetic fertilisers [1]. In Ivory Coast, tilapia (*Oreochromis niloticus*) aquaculture represents a key source of animal protein; however, intensive production practices and the widespread use of antibiotics to manage bacterial diseases increasingly drive antimicrobial resistance (AMR), threatening both food safety and ecosystem health [2,3].

Probiotic lactic acid bacteria (LAB) represent an ecologically sound and economically viable alternative to antibiotics in aquaculture, improving feed conversion, stimulating innate immunity, and enhancing water quality [4,5,6,7]. Strains of the genus *Enterococcus*, despite their dual identity as commensals and opportunistic pathogens, are frequently identified as dominant members of the fish-gut microbiome and have been extensively characterised for multifunctional probiotic properties [8,9]. The safety assessment of *Enterococcus*-based probiotics, in particular the absence of transferable antibiotic resistance genes and virulence determinants, is therefore a prerequisite for responsible application [10]. In a preceding phenotypic screening conducted by our group [11], twelve LAB isolates from a tilapia aquaculture pond at the University Nangui Abrogoua, Ivory Coast were screened for probiotic, PGP, and nitrifying properties. Three isolates, LAB1, LAB10, and LAB11, emerged as the most functionally relevant; LAB1 and LAB10 demonstrated the highest ammonia-oxidising capacity both in vitro and in trout pond water, while LAB11 was the sole isolate to produce indole-3-acetic acid (IAA; 17.72 ± 0.06 μg/mL), siderophores, and solubilise phosphate. All three strains were identified by 16S rDNA sequencing as *Enterococcus faecalis*. None exhibited haemolytic activity.

Although LAB1, LAB10, and LAB11 have been characterised phenotypically for probiotic, PGP, and nitrifying activity, the genetic basis underlying these traits, their relationship to publicly available *E. faecalis* genomes, and their safety at the whole-genome level remain undefined, a critical gap prior to any applied use of these strains in aquaponic systems. To address this gap, we sequenced, assembled, and annotated the draft genomes of LAB1, LAB10, and LAB11. Here we report these draft genome sequences, together with MLST, antibiotic resistance gene (ARG) screening, virulence factor analysis, pan-genome comparison against the reference strain and related *Enterococcus* species, and identification of functional gene content associated with the probiotic, PGP, and nitrifying properties of these strains. To our knowledge, this is the first genomic characterisation of *E. faecalis* isolates from Ivory Coast and a first from West African tilapia aquaponic system that combines probiotic, plant growth-promoting, and heterotrophic nitrifying phenotypes with whole-genome analysis, providing a genomic resource not represented among existing, predominantly clinical- or dairy-derived, *E. faecalis* reference genomes.

## 2. Materials and Methods

### 2.1. Bacterial Strains and Culture Conditions

Strains LAB1, LAB10, and LAB11, previously identified as *Enterococcus faecalis* by 16S rDNA sequencing (GenBank accessions PX518070, PX518071, and PX518072, respectively), were isolated from pond water of the tilapia aquaculture facility at the University Nangui Abrogoua, Abidjan, Ivory Coast. Working cultures for DNA extraction were prepared by two successive transfers in de Man, Rogosa and Sharpe (MRS) broth (Biokar Diagnostics, Beauvais, France) at 37 °C, 150 rpm, for 48 h.

### 2.2. DNA Extraction, Sequencing, and Assembly

Genomic DNA was extracted from overnight cultures using bacterial genomic DNA extraction Qiagen Kit (Liege, Belgium) according to the manufacturer’s instructions. DNA purity and concentration were assessed by NanoDrop^TM^ (Thermo Fischer Scientific, Wilmington, DE, USA). Paired-end sequencing libraries were prepared and sequenced on an Illumina platform at Novogene Europe. Raw reads were quality-filtered and trimmed using Trimmomatic v0.39 (minimum Phred Q30) [12]. De novo assembly was performed using SPAdes v4.2 [13] with default k-mer parameters. Assembly quality was evaluated using QUAST v5.3.0 [14].

### 2.3. Genome Annotation

Genome annotation was performed using Prokka v1.15.6 [15] with the default bacterial annotation mode against the UniProtKB/Swiss-Prot database. Functional categorisation of predicted proteins was performed by assignment to Clusters of Orthologous Groups (COGs) via eggNOG-mapper v2.1.13 [16] against the eggNOG 5.0 database. Gene ontology (GO) enrichment analysis of shared gene clusters was performed using the goatools Python library v1.6.5 [17]. To cross-validate the Prokka-based gene calls and to check for additional genes relevant to the probiotic, plant growth-promoting, and nitrogen-cycling phenotypes, the three genome assemblies were independently re-annotated with Bakta v1.12.0 (database v6.0, light) [18] and DFAST [19], both run with default parameters. Gene models from all three pipelines (Prokka, Bakta, DFAST) were cross-compared; results are summarised in Table 1.

### 2.4. Multilocus Sequence Typing

MLST was performed in silico using the staramr tool (v0.12.1) [20] with the PubMLST *Enterococcus faecalis* scheme, which targets seven housekeeping loci (gdh, gyd, pstS, gki, aroE, xpt, yiqL).

### 2.5. Functional Gene Analysis and Pan-Genome

Key functional genes associated with probiotic, PGP, and nitrogen-cycling properties were identified through Prokka annotations and BLASTp searches against UniProtKB/Swiss-Prot (identity ≥ 40%, e-value ≤ 10^−15^). Plant growth-promoting traits were predicted using the PGPT-Pred module of the PLaBAse web resource (v1.01; https://plabase.cs.uni-tuebingen.de/pb/plabase.php (accessed on 1 April 2026)) [21], which annotates bacterial protein sequences against reference sequences. Results were filtered to retain annotations with e-value ≤ 10^−10^ and cross-referenced against Prokka functional annotations and COG categories for consistency. Pan-genome analysis was performed using OrthoVenn3 and a custom clustering pipeline with the reference strain *Enterococcus faecalis* V583 (GenBank AE016830). Orthologous groups were defined by an all-against-all DIAMOND BLASTp search (e-value ≤ 10^−5^) followed by clustering with the default Markov clustering algorithm (inflation value 1.5). GO enrichment of shared gene clusters was evaluated with a false discovery rate (FDR) threshold of 0.05. Whole-genome phylogenomic relationships were inferred from a concatenated alignment of shared single-copy core gene clusters identified by OrthoVenn3. The alignment was analysed by maximum likelihood using IQ-TREE v2.2.0 with the best-fit substitution model (LG + F + G4) and 1000 ultrafast bootstrap replicates.

### 2.6. Antibiotic Resistance Gene and Plasmid Screening

Antibiotic resistance genes (ARGs) were predicted using staramr v0.12.1 against the ResFinder database [22]. Plasmid replicon typing was performed using PlasmidFinder 2.1 [23], integrated within the staramr pipeline.

### 2.7. Virulence Factor Analysis

Virulence factors were identified using ABRICATE v1.0.1 against the Virulence Factor Database (VFDB) [24], with a minimum nucleotide identity threshold of 98% and minimum coverage of 98%.

### 2.8. Probiotic Probability Scoring

Per-contig probiotic probability scores were computed using ProbML [25], ProbML was trained on curated sets of probiotic and non-probiotic prokaryotic genomes and benchmarked against five machine-learning algorithms; XGBoost achieved the highest accuracy, attaining 100% on the training dataset and 95.45% on an independent test dataset, outperforming existing probiotic genome classification tools. Each contig (genome node) per isolate was individually submitted to the classifier, which outputs a continuous probability score in the range [0, 1], where values approaching 1 indicate high confidence in probiotic classification.

## 3. Results

### 3.1. General Genome Features and MLST

The general genomic features of LAB1, LAB10, and LAB11 are summarised in Table 1. SPAdes assembly of Illumina paired-end reads yielded draft genomes with few ambiguous bases (N’s per 100 kbp: 3.26, 6.59, and 9.85 for LAB1, LAB10, and LAB11, respectively). LAB1 comprises 34 contigs (2,944,145 bp; N50 = 248,560 bp; GC = 37.40%), LAB10 comprises 35 contigs (2,944,827 bp; N50 = 248,560 bp; GC = 37.40%), and LAB11 comprises 34 contigs (2,943,091 bp; N50 = 247,803 bp; GC = 37.40%). The largest contig (376,685 bp) is identical across all three assemblies. Prokka annotation predicted 2872 CDS, 4 rRNA, 54 tRNA, and 1 tmRNA for LAB1; 2873 CDS, 4 rRNA, 54 tRNA, and 1 tmRNA for LAB10; and 2875 CDS, 4 rRNA, 54 tRNA, and 1 tmRNA for LAB11. In silico MLST (efaecalis scheme) assigned all three strains to sequence type ST19. Hypothetical proteins account for approximately 39–40% of predicted CDS per genome (LAB1: 1150; LAB10: 1151; LAB11: 1152).

**Table 1:** Genomic characteristics of *Enterococcus faecalis* strains LAB1, LAB10, and LAB11.

Genomic Feature	LAB1	LAB10	LAB11
Species (16S rDNA)	*E. faecalis*	*E. faecalis*	*E. faecalis*
MLST	ST19	ST19	ST19
GenBank 16S accession	PX518070	PX518071	PX518072
Sequencing platform	Illumina	Illumina	Illumina
Total assembly size (bp)	2,944,145	2,944,827	2,943,091
Number of contigs	34	35	34
Largest contig (bp)	376,685	376,685	376,685
N50 (bp)	248,560	248,560	247,803
N90 (bp)	69,651	69,651	69,651
GC content (%)	37.40	37.40	37.40
N’s per 100 kbp	3.26	6.59	9.85
Protein-coding sequences (Prokka)	2872	2873	2875
Hypothetical proteins (Prokka)	1150	1151	1152
rRNA genes (Prokka)	4	4	4
tRNA genes (Prokka)	54	54	54
tmRNA genes (Prokka)	1	1	1
Protein-coding sequences (Bakta)	2872	2873	2875
rRNA genes (Bakta)	5	5	5
tRNA genes (Bakta)	57	57	57
tmRNA genes (Bakta)	1	1	1
CRISPR (Bakta)	2	1	1
Protein-coding sequences (DFAST)	2893	2899	2897
rRNA genes (DFAST)	1	1	1
tRNA genes (DFAST)	54	54	54
CRISPR (DFAST)	2	2	2
GenBank WGS accession	JBXPLQ000000000	JBXPLP000000000	JBXPLO000000000

### 3.2. Pan-Genome Analysis and Phylogenomics

**Figure 1:**
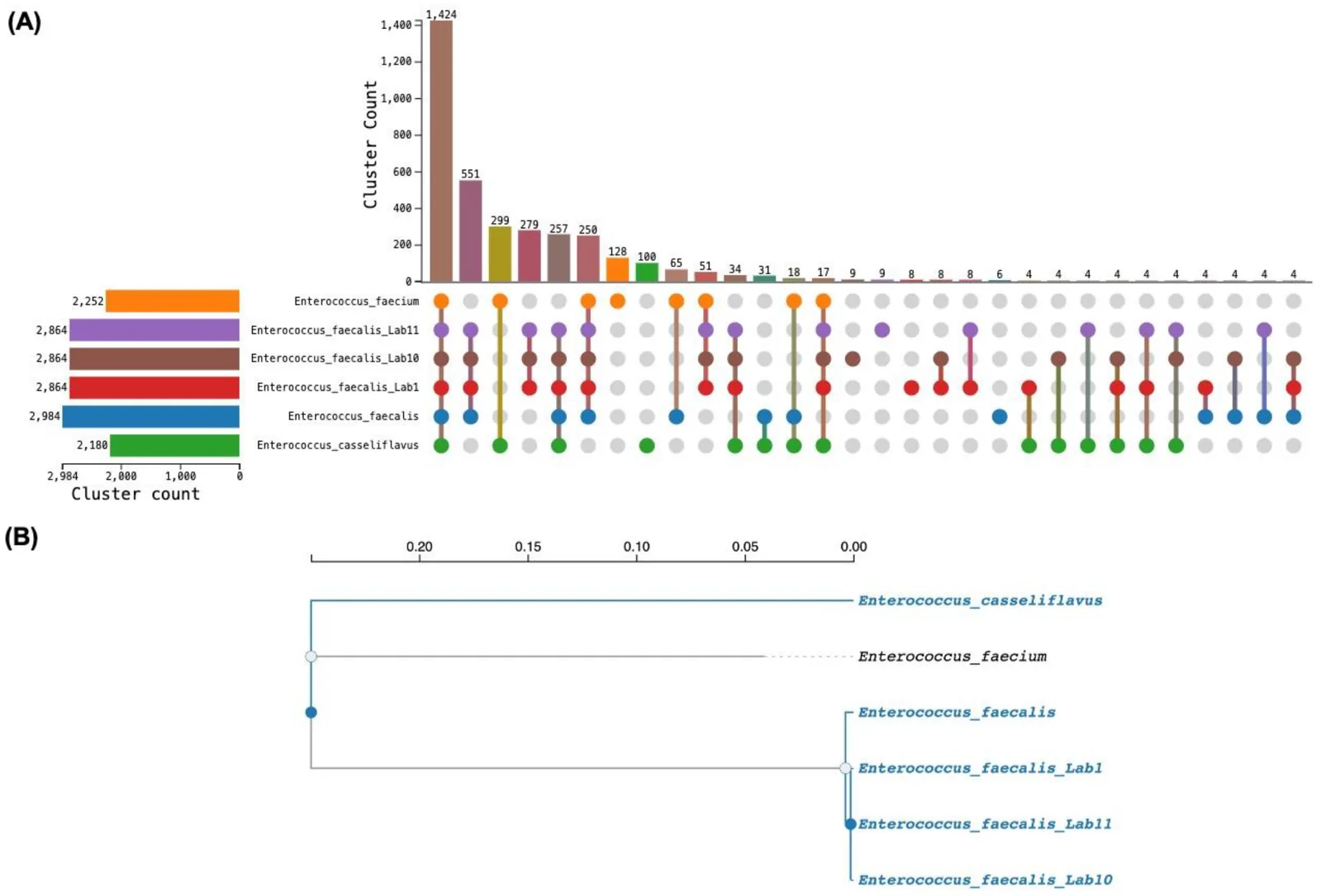
Pan-genome analysis and whole-genome phylogenomics of *Enterococcus faecalis* LAB1, LAB10, and LAB11. (**A**) UpSet plot of gene-cluster distribution across six genomes: *E. faecalis* LAB1, LAB10, and LAB11; *E. faecalis* V583; *E. faecium*; and *E. casseliflavus*. Horizontal bars (left) show total gene-cluster content per genome; vertical bars (top) show the count of clusters at each intersection. The 1424 clusters shared by all six taxa constitute the core genome while 279 clusters are exclusive to the three isolates relative to the reference strains. (**B**) Whole-genome phylogenomic tree inferred from shared single-copy gene clusters. Branch lengths are in substitutions per site. Branch colour depicts group assignment only, branches whose descendants all belong to a single defined group are coloured blue, while branches with descendants from more than one group is coloured black.

A pan-genome comparison was performed among LAB1, LAB10, LAB11, and the reference strains *E. faecalis* V583, *E. faecium* Aus0004, and *E. casseliflavus* EC20. Gene-cluster presence/absence across the six genomes was visualised in Figure 1A. Across the six genomes, 6504 orthologous gene clusters were identified. The pan-genome of the four *E. faecalis* species comprised 3935 clusters, of which 551 clusters (14.0%) formed the strict core genome shared by all four isolates. Gene ontology enrichment analysis of the 551 universal core clusters highlighted functions central to *E. faecalis* biology and host–microbe interactions (FDR ≤ 0.05). The most significantly enriched biological process was GO:0009401 (phosphoenolpyruvate-dependent sugar phosphotransferase system) with an extreme *p*-value of 1.43 × 10^−44^. Other enriched terms included GO:0006355 (regulation of transcription, DNA-templated) (*p* = 6.34 × 10^−18^), GO:0043571 (maintenance of CRISPR repeat elements) (*p* = 4.47 × 10^−8^), and GO:0032196 (transposition) (*p* = 2.55 × 10^−5^), signifying that genome plasticity and phage defence are conserved features of this group.

Each of the three strains possessed 2864 gene clusters, slightly fewer than *E. faecalis* V583 (2984 clusters). When comparing the three LAB isolates directly to V583, 279 gene clusters were found exclusively in our strains and absent from V583 (Figure 1A). Pairwise comparison showed that all 279 gene clusters are shared by all three strains (Figure 1B). One gene cluster is shared exclusively by LAB10 and LAB11, with no clusters unique to any single strain. The 279 gene clusters were enriched in GO:0006807 (nitrogen compound metabolic process; 13 genes), GO:0044238 (primary metabolic process; 9 genes), and GO:0006725 (cellular aromatic compound metabolic process; 10 genes), traits commonly associated with metabolic adaptation to the nutrient-rich, nitrogen-cycling pond environment. The presence of a complete CRISPR-Cas system (*cas9*, *cas1*, *cas2*, *csn2*) within the exclusive gene set provides molecular evidence for active phage–host co-evolution in the aquaculture microbiome. Independent re-annotation with Bakta and DFAST identified two further genomic differences among the three isolates that fall outside standard ortholog clustering and were not captured in the pan-genome analysis above. First, LAB1 carries a second, cas-gene-free CRISPR repeat array (a distinct four-repeat locus with a different consensus repeat sequence from the shared type II-A array described above) that is absent from LAB10 and LAB11. Second, the DFAST pseudogene caller identified the *E. faecalis* collagen-binding MSCRAMM adhesin gene (ace) as disrupted by a frameshift in LAB11, whereas the same locus is annotated as a single intact coding sequence in LAB1 and overlaps an assembly gap in LAB10; because all three draft genomes retain a small number of unresolved bases at this stage (Table 1), we cannot yet distinguish genuine gene loss from an assembly artefact at this specific locus, and confirmation would require targeted gap closure or long-read resequencing.

Whole-genome phylogenomic analysis (Figure 1B) placed all three LAB strains within the *Enterococcus faecalis* clade, clustering with *E. faecalis* V583. LAB1 and LAB11 shared a terminal node, while LAB10 diverged marginally, likely due to the single gene-cluster difference detected in the pan-genome comparison. The outgroup species *E. faecium* and *E. casseliflavus* formed distinct lineages, confirming accurate taxonomic placement.

### 3.3. Safety Genomics: Antibiotic Resistance Genes and Virulence Factors

Screening against the ResFinder/staramr database identified two acquired antimicrobial resistance genes in all three strains: *tet(M)* (100% coverage, 100% nucleotide identity) and *lsa(A)* (98.4% coverage, 100% nucleotide identity). Both genes were co-located on a contig that also carries the repUS43 plasmid replicon (99.83% identity to CP003584). No vancomycin resistance genes (*vanA*, *vanB*, or *vanC*) were detected in any of the three genomes. VFDB screening identified 14 virulence factor homologs conserved across all three strains (Table 2), with ≥98.48–99.94% nucleotide identity to *E. faecalis* V583: capsule biosynthesis genes (*cpsA*, *cpsB*), hyaluronidase (*EF0818*), the Fsr quorum-sensing system (*fsrB*, *fsrC*), gelatinase (*gelE*), serine proteinase (*sprE*), the *Ebp* pilus operon (*ebpA*, *ebpB*, *ebpC*, *srtC*), endocarditis antigen (*efaA*), transcriptional regulator (*bopD*), and fibrinogen-binding surface protein (*fss1*). No cytolysin operon genes (*cylA-M*) were detected in any genome.

### 3.4. Genomic Basis for Probiotic Properties

Genome annotation using Bakta, Prokka, and Dfast identified sets of functional genes in all three genomes that are consistent with, and provide a plausible mechanistic contribution to, several of the probiotic phenotypes summarised in Table 3. “Plausible” because gene presence is not itself proof of the causal mechanism, and confirmation of these links would require targeted enzymatic assays or gene-knockout experiments. Antioxidant capacity, expressed as DPPH radical-scavenging activity of 67.60–78.40% in cell-free culture supernatants, co-occurs with three oxidative stress resistance genes, namely katA (vegetative catalase), ydbD (putative Mn-type catalase), and sodA (Mn-dependent superoxide dismutase). Tolerance to bile salts, quantified as 2.13–40.87% relative growth at 0.3% conjugated bile salts, is consistent with the presence of *cbh*, encoding choloylglycine hydrolase (bile salt hydrolase, BSH). Beyond the production of organic acids, the broad-spectrum antibacterial activity of extracellular supernatants from the LAB isolate against *Escherichia coli* ATCC 25922, *Staphylococcus aureus* ATCC 29213, *Pseudomonas aeruginosa* ATCC 27853, and *Aeromonas hydrophila* is genetically supported by the *uviB* gene, which is associated with bacteriocin biosynthesis. Cross-annotation with Bakta and DFAST expanded this genomic picture beyond the single *uviB* call, identifying a small bacteriocin-associated gene set present in all three genomes: an Enterocin A immunity protein, a separate bacteriocin immunity protein, a bacteriocin-family protein, and a *ComC/BlpC*-family peptide pheromone precursor typical of class II bacteriocin quorum-sensing regulation in lactic acid bacteria. Survival under simulated gastric conditions (0.3% pepsin, pH 1.5) has a clear genomic complement in the heat shock chaperone genes *groEL*, *groES*, and *dnaK*. The complete absence of the cytolysin locus (*cylA-cylM*) in all three assemblies is fully consistent with the gamma-haemolysis phenotype confirmed phenotypically in the preceding screening [11]; since cytolysin is the primary pore-forming toxin responsible for beta-haemolysis in virulent *E. faecalis*, its absence eliminates a major biosafety concern [26]. Finally, the Ebp pilus operon (*ebpA*, *ebpB*, *ebpC*, *srtC*), surface adhesin *efaA*, and fibrinogen-binding protein fss1 collectively encode a surface attachment mechanism consistent with the moderate cell-surface hydrophobicity values measured (chloroform, 5.50–22.83%; hexane, 0.83–13.16%) using wet lab assays by Coulibaly et al. [11].

**Figure 2:**
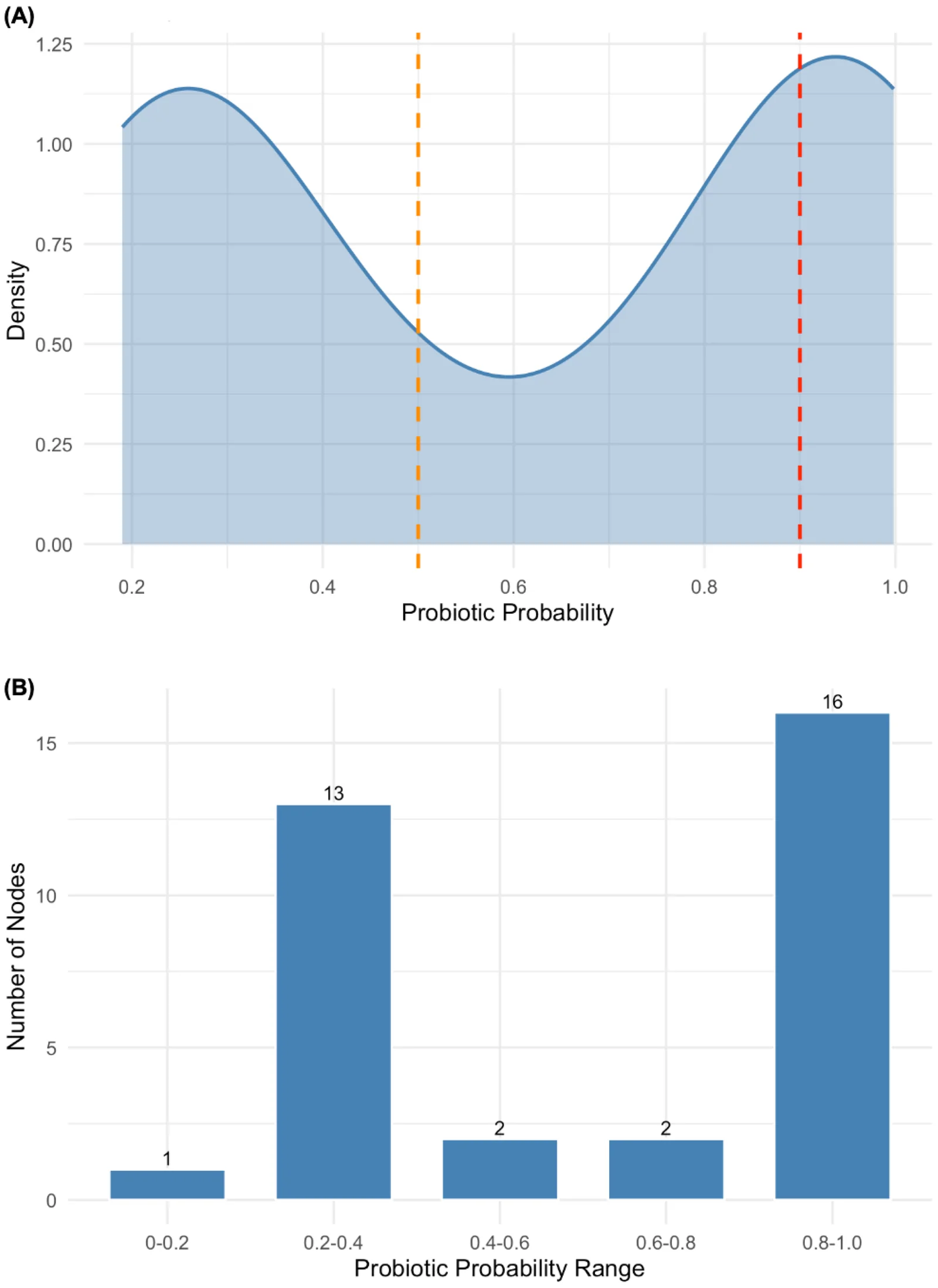
ProbML probiotic probability score distribution for *Enterococcus faecalis* LAB1, LAB10, and LAB11. Because the three assemblies are clonally identical, their score distributions were indistinguishable, and the figure is representative of all three strains. (**A**) Histogram (bin width = 0.05) of per-contig probiotic probability scores. Dashed lines mark the 0.5 decision threshold (orange) and 0.9 high-confidence threshold (red). (**B**) Count of contigs by probiotic probability bin. A total of 16 contigs fall in the 0.80–1.00 range (chromosomal) and 13 contigs in the 0.20–0.40 range (extrachromosomal); only 4 contigs fall in intermediate bins (0.40–0.80).

Independent, genome-wide corroboration of the probiotic potential was obtained using ProbML [25]. Per-contig scores for all three strains are reported in Table S1; because the three assemblies are clonally identical in gene content, their score distributions were indistinguishable. Across all three genomes, contigs fell into two scoring clusters: 16 contigs received high probiotic probability scores (0.80–1.00), corresponding to the main chromosomal backbone, while 13 contigs clustered in the 0.20–0.40 range, corresponding to smaller, high-coverage contigs consistent with plasmid or mobile genetic element sequences (Figure 2). The bimodal distribution, with 38.2% of contigs scoring above 0.90 and a clear gap between the two clusters, confirms that the chromosomal backbone of all three strains carries a strong genomic signature characteristic of recognised probiotic microorganisms, while the lower-scoring contigs bearing tet(M) and the repUS43 replicon are appropriately distinguished by the classifier as non-chromosomal elements inconsistent with the probiotic signature. Taken together, the gene-level analysis and the ProbML classification converge on the same conclusion, that the chromosomal architecture of LAB1, LAB10, and LAB11 is consistent with a functional probiotic organism, with the tet(M)-carrying plasmid representing the sole outstanding safety qualification.

### 3.5. Genomic Determinants of Plant Growth-Promoting Activity

Based on the phenotypic PGP profiling, LAB11 was the sole IAA producer among the twelve isolates screened (Coulibaly et al. [11]), yielding 17.72 ± 0.06 µg/mL, as determined colorimetrically using the Salkowski reagent method (filter-paper qualitative screening followed by quantification against a 5–100 µg/mL standard curve at 536 nm, as detailed in the companion phenotypic screening study; Coulibaly et al. [11]). Application of a 1:50 dilution of LAB11 cell-free supernatant significantly enhanced maize germination rate (86.66 ± 5.77% versus 63.33 ± 32.14% for the uninoculated control; *p* < 0.05) and radicle length (7.00 ± 0.52 mm versus 4.94 ± 1.52 mm; *p* < 0.05), than the uninoculated control. We note that the control group showed high inter-replicate variability (SD = 32.14 percentage points for germination rate), such that the numerical ranges of the two groups overlap; although the applied test indicated a significant difference between means, this variance limits confidence in the true effect size, and the result should be treated as preliminary evidence of biostimulant activity. Genomic analysis did not identify the canonical IAA biosynthesis genes ipdC (indole pyruvate decarboxylase) or tso (tryptophan side-chain oxidase) in any of the three assemblies. The genomes did contain tryptophanyl-tRNA synthetase genes (*trpS*, *trpS2*) and the tRNA isopentenyltransferase miaA; however, these are non-specific housekeeping genes required for protein synthesis and tRNA modification in all bacteria, are not exclusive to IAA metabolism, and their presence cannot be taken as evidence for any particular IAA biosynthetic route. We therefore cannot identify a specific genomic pathway underlying IAA production in LAB11 from the present annotation; alternative pathways yielding IAA from tryptophan have been documented in LAB in the absence of canonical genes [27]. Siderophore production was confirmed phenotypically for LAB1 (64.45 ± 0.19 SU%), LAB10 (73.91 ± 0.56 SU%), and LAB11 (73.02 ± 0.61 SU%) on CAS. Despite this capacity, no siderophore biosynthesis genes were identified in any assembly, and the ferric-siderophore import genes *fepC* (ferric enterobactin transport ATPase) and *fhuD* (iron(3+)-hydroxamate-binding protein) detected in all three genomes encode uptake of siderophores synthesised by other organisms rather than siderophore biosynthesis, and therefore do not explain the observed CAS-positive phenotype. We do not consider the available genomic data to support the siderophore production phenotype; the molecular basis of this activity remains unresolved and could be due to an unannotated or divergent biosynthetic gene cluster not captured by the reference-based annotation used here. Resolving this discrepancy will require further research with dedicated genome mining beyond homology-based annotation, chemical characterisation of the chelating compound, or both.

All isolates solubilised inorganic phosphate on NBRIP agar, producing visible halo zones of 12.66 ± 2.08 mm to 20.33 ± 0.57 mm diameter; however, no potassium solubilisation was observed under the potassium-adapted variation in the same assay conditions. All three genomes encode four phosphatase genes identified by Prokka, namely *acyP* (acylphosphatase), *uppP* (undecaprenyl pyrophosphate phosphatase), *gph* (phosphoglycolate phosphatase), and *pspA* (phage shock protein A, phosphatase domain); cross-annotation with Bakta and DFAST additionally identified two alkaline phosphatase-family genes (one annotated as a PhoP-regulated alkaline phosphatase, the other an unlinked alkaline phosphatase paralogue) present in all three genomes but not separately called by Prokka, together with an intact *PstS/PhoU* phosphate-specific transport (*Pst*) operon. The expression of gene encoding phosphatase enzymes we found break down organic phosphorus compounds and cell membranes; they do not dissolve hard mineral phosphates. The NBRIP assay, on the other hand, tests for the ability to dissolve a specific hard mineral (tricalcium phosphate), which usually happens through the secretion of organic acids. None of the three gene-prediction tools we used contained the genes needed to produce the required acids in any of our strains. Therefore, the phosphatase genes we identified cannot explain the clearing zones we saw on the NBRIP plates.

All isolates were also positive for ammonia production (Nessler assay), and ammonia-related metabolic genes (*eutB*, *eutC*, *gdhA*) are present in all three assemblies. Bakta annotation confirmed *eutB/eutC* (as the ethanolamine ammonia-lyase large and small subunits) and *gdhA* (glutamate dehydrogenase) in all three genomes, and additionally identified an ammonium transporter (Amt-family) and a diaminopropionate ammonia-lyase, both present in all three assemblies, which provide further plausible routes for ammonia generation and assimilation.

**Figure 3:**
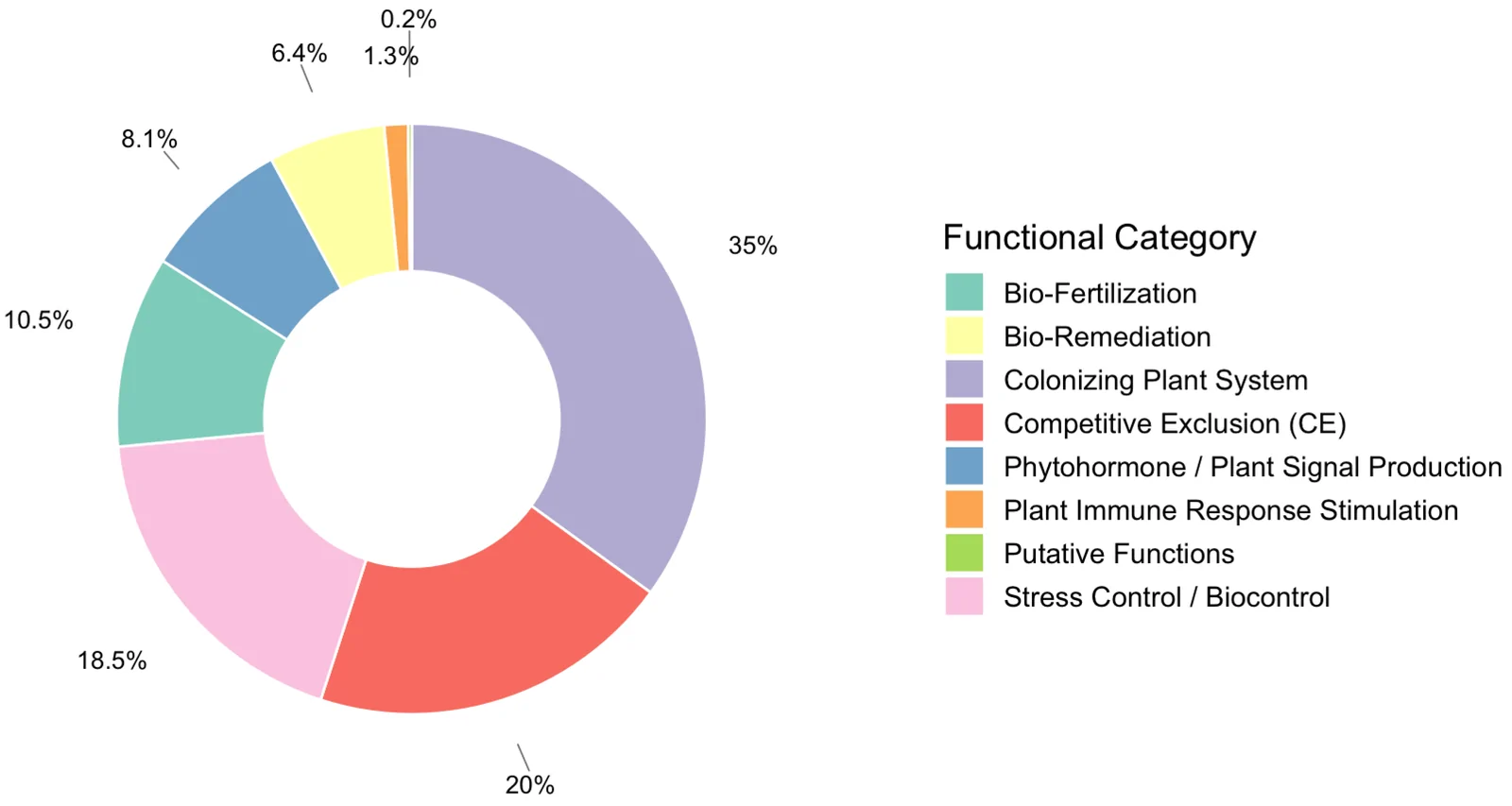
Pie chart showing the distribution of approximately 994–995 predicted plant growth-promoting gene associations across eight functional categories in *Enterococcus faecalis* LAB1, LAB10, and LAB11. All three isolates showed an identical category distribution.

**Figure 4:**
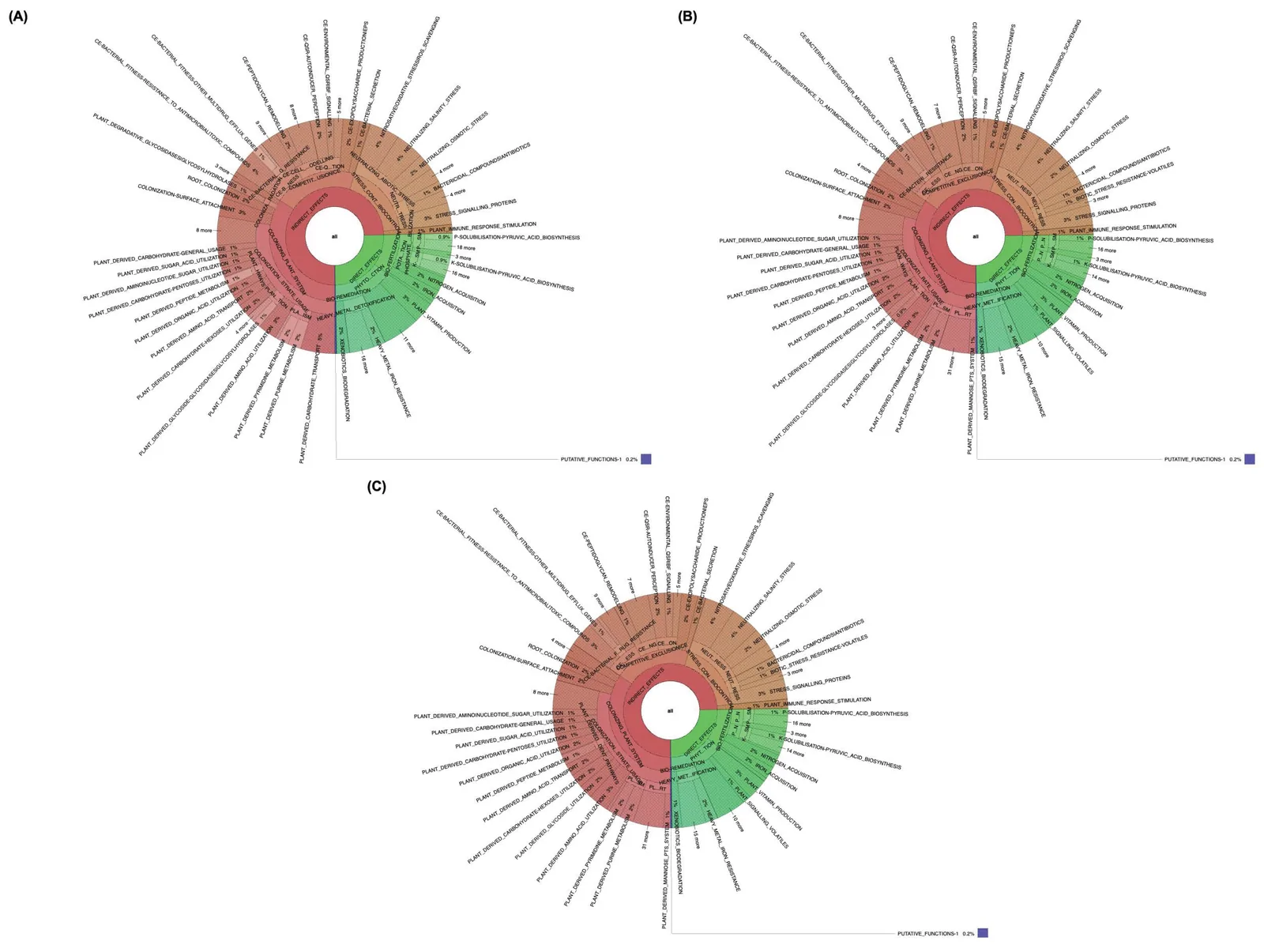
Krona charts showing the full annotation hierarchical distribution from top-level functional categories to specific biochemical pathways of predicted plant growth-promoting traits (PGPTs) for (**A**) LAB1, (**B**) LAB10, and (**C**) LAB11, generated from PLaBAse v1.01.

PLaBAse PGPT-Pred analysis identified 994–995 PGP trait gene associations per genome across eight functional categories. The category distribution identical across all three isolates were: Colonising Plant System (34.6–34.7%), Competitive Exclusion/CE (20.3–20.4%), Stress Control/Biocontrol (18.5%), Bio-Fertilisation (10.5%), Phytohormone/Plant Signal Production (8.1%), Bio-Remediation (6.4%), Plant Immune Response Stimulation (1.3%), and Putative Functions (0.2%) (Figure 3). Within Bio-Fertilisation, the principal subcategories were phosphate solubilisation (94 hits), potassium solubilisation (74), nitrogen acquisition (49), and iron acquisition (42). Interestingly, none of the isolates showed phenotypic potassium solubilisation even though PLaBAse PGPT-Pred assigned 74 hits to a potassium solubilisation subcategory; we hypothesise that this is likely reflecting annotation of genes with peripheral roles in potassium transport rather than direct mineral dissolution. The Colonising Plant System category was dominated by plant-derived substrate utilisation genes (755 hits per genome), with additional contributions from surface attachment (49) and root colonisation (43) genes. The hierarchical visualisation in Figure 4 revealed the full breadth of annotated pathways within each category.

### 3.6. Genomic Basis for Nitrifying Capacity

All isolates produced ammonia and showed measurable in vitro ammonia oxidation (0.70–1.54 g/L). LAB10 exhibited the highest nitrifying capacity, producing nitrite at 0.35 ± 0.04 mg/L and nitrate at 8 mg/L in vitro; LAB1 was second in nitrate production at 4 mg/L. In a trout pond water validation experiment, inoculation with LAB1 resulted in nitrate accumulation of 110 mg/L (ammonia: 6.4 mg/L; nitrite: 8.8 mg/L), while LAB10 produced nitrate at 80 mg/L (ammonia: 8.8 mg/L; nitrite: 16 mg/L), confirming that both strains drive net ammonia oxidation in a realistic aquatic matrix. Despite this documented ammonia-oxidising phenotype, classical autotrophic ammonia monooxygenase genes (*amoA*, *amoB*, *amoC*) were absent from all three assemblies, excluding chemoautotrophic nitrification as the underlying mechanism. The presence of *eutB* and *eutC* (ethanolamine ammonia-lyase, large and small subunits) in all three genomes (Table 3) offers one plausible heterotrophic route that could contribute to ammonia turnover; glutamate dehydrogenase (*gdhA*), also present in all three assemblies, could similarly contribute to oxidative deamination of glutamate to alpha-ketoglutarate with ammonia release, and may additionally serve to assimilate free ammonia under nitrogen-replete conditions; as with *eutB/eutC*, this remains a genomic inference rather than an experimentally demonstrated mechanism.

## 4. Discussion

The genome assemblies generated for LAB1, LAB10, and LAB11 meet the quality thresholds for downstream functional and comparative genomic analyses. Genome sizes of 2.943–2.945 Mb with a GC content of 37.40% fall comfortably within the range documented for *E. faecalis*; the type strain used in this study (V583) measures 3.22 Mb with 37.5% GC [28] and the low ambiguity base rates indicate near-complete chromosomal coverage despite the short-read draft format. MLST assignment to ST19 places all three isolates within one of the most widely distributed clonal lineages of *E. faecalis*, a sequence type recovered from gastrointestinal tracts, food products, livestock, and environmental water samples across multiple continents [29]. The identification of ST19 in West African aquaculture ponds therefore broadens its known ecological range. Perhaps the most striking finding of the comparative genomic analysis is the near-complete genetic identity of the three strains. With 2864 genes each, no strain-unique gene clusters, and only a single cluster differentiating LAB10/LAB11 from LAB1, the three isolates almost certainly represent a clonal population descended from a single ancestral colonisation event within the tilapia pond. Given this near-clonal relationship, we considered whether LAB1, LAB10, and LAB11 should be reported as a single strain rather than three separate genomes; we retained them as three separately deposited assemblies because they were isolated as independent colonies in the preceding phenotypic screening and displayed measurable, reproducible differences in nitrifying capacity and IAA production (Table 3), and because the minor differences in contig structure, N50, and the single differentiating gene cluster may reflect genuine within-population microevolution that merits preservation in the public record rather than an assembly artefact. Cross-annotation with Bakta and DFAST provided two further lines of evidence for minute genuine divergence among the isolates beyond assembly noise: an additional, cas-gene-free CRISPR repeat array unique to LAB1, and a possible disruption of the collagen-adhesin gene ace in LAB11, both indicating the isolates having accumulated small, strain-specific differences since diverging from a common ancestor. A parallel 2026 study of *E. faecalis* isolated from the gut of the flatfish *Solea solea* similarly reported ST19 among the dominant sequence types, with probiotic gene traits comparable to those documented in this study; this suggests that ST19 may represent a recurrent, well-adapted commensal genotype across diverse aquaculture systems [30].

The presence of 14 virulence factor homologs identified against the Virulence Factor Database is important. These homologs include the entire *Ebp* pilus operon (*ebpA*, *ebpB*, *ebpC*, *srtC*), the endocarditis-specific antigen *efaA*, the collagen-binding adhesin *fss1*, and the *Fsr* quorum-sensing system that regulates gelatinase (*gelE*) and serine protease (*sprE*). Capsular biosynthesis genes *cpsA* and *cpsB* and the hyaluronidase EF0818 were also detected. Our analysis indicates that these isolates retain the genetic potential for opportunistic pathogenicity in both humans and animals, particularly if they translocate from the gut to sterile body organs. This risk is substantially mitigated by the absence of cytolysin locus (*cylA-M*); we consider this the single most important indicator of reduced virulence potential. Cytolysin is a pore-forming toxin that lyses eukaryotic cells and erythrocytes, and its expression in clinical *E. faecalis* strains has been associated with a five-fold increase in the probability of an acutely fatal outcome [26]; its absence is fully consistent with the gamma-haemolysis phenotype documented for all three isolates. This finding is supported by a study screening *E. faecalis* from food and clinical isolates and found the *cyl* operon absent in approximately 80% of dairy and environmental strains, with beta-haemolysis correlating with *cyl* operon presence rather than suppressed expression [31]. Another directly comparable observation was reported in *E. faecalis* CAUM157, a bacteriocinogenic strain recently advanced as a probiotic candidate specifically on the basis of *cyl* operon absence, gamma-haemolysis, and vancomycin susceptibility [32].

In addition, the *Ebp* pilus operon (*ebpA*, *ebpB*, *ebpC*, *srtC*), surface adhesin *efaA*, and fibrinogen-binding protein *fss1* primarily mediate attachment to epithelial surfaces, capabilities that, in a probiotic context, translate to competitive mucosal colonisation and pathogen exclusion. However, the *Fsr* quorum-sensing system (*fsrB*, *fsrC*) and its regulated target gelatinase (*gelE*) merit continued monitoring, since gelatinase activity has been linked to virulence in immunocompromised hosts, but its relevance in healthy fish or aquaculture applications is expected to be negligible. The most important genomic safety concern is the co-occurrence of *tet(M)* and the repUS43 plasmid replicon across all three isolates. The *tet(M)* gene is a ribosome protection protein and is commonly associated with conjugative transposons of the Tn916/Tn1545 family [33]. Although no conjugation (*tra*) operon was detected in the current assembly, the possibility of mobilisation in trans by a co-resident conjugative element elsewhere in the genome cannot be excluded. By contrast, *lsa(A)* is an intrinsic ABC efflux gene whose intact coding sequence is a species-defining characteristic of *E. faecalis*; it confers natural low-level resistance to lincosamides and streptogramins A and is not considered a transferable resistance risk [34]. The complete absence of *vanA*, *vanB*, and *vanC* is a critical safety-positive finding since vancomycin resistance represents an absolute contraindication to probiotic application under EFSA and FAO/WHO evaluation frameworks [11]. Our safety genomics picture is consistent with the Qualified Presumption of Safety (QPS) framework, with the *tet(M)*-carrying plasmid remaining the clear priority for further characterisation.

Alongside their safety characteristics, the isolates were also screened for their potential to survive in the gastrointestinal tract and confer health benefits. We identified genomic determinants of experimentally observed antioxidant, bile salt tolerance, and antibacterial activities. Both *katA* (catalase) and *sodA* (Mn-dependent superoxide dismutase) were identified across all three assemblies, which together provide a mechanistic basis for the high DPPH radical-scavenging activity measured. The co-expression of these two enzymes is established as the principal driver of oxidative stress resistance in LAB, and their release accounts for extracellular antioxidant activity measurable in spent culture supernatants [35]. This DPPH radical-scavenging activity measured in this study is higher than the 41.65% DPPH scavenging reported for *E. durans* isolates in a LAB probiotic panel, and are consistent with the superior antioxidant capacity reported for marine-derived *E. faecalis* HY0110, where the strain showed greater free-radical scavenging than the benchmark probiotic *Lactobacillus rhamnosus* GG in direct comparative assays [36]. Bile salt tolerance is similarly explained by a cbh-encoded bile salt hydrolase. BSH-mediated deconjugation of primary bile acids reduces their antimicrobial potency and is recognised as a key determinant of intestinal persistence in probiotic LAB [37]. However, it should be noted that BSH activity is not exclusively beneficial; a recent comprehensive review of microbial BSH biology highlighted that, in *E. faecalis* specifically, cbh-encoded BSH can enhance bacterial persistence and systemic dissemination in immunocompromised hosts, functioning as a dual probiotic–virulence attribute [38]. A context-dependent consideration is therefore necessary when evaluating these strains for application in immunologically vulnerable fish populations.

The bacteriocin biosynthesis gene uviB provides a genomic basis for the antibacterial activity isolates. The production of enterocins by *E. faecalis* from aquatic and food environments was also reported in *E. faecalis* Gr17, which was isolated from a Chinese fermented fish product; the study reported by genetic and biochemical evidence that the isolate produced a novel Sec-dependent enterocin with inhibitory activity against a range of food-borne pathogens [24]. In another study, bacteriocinogenic probiotic bacteria isolated from freshwater aquatic environments were shown to inhibit the growth of *Aeromonas hydrophila* and other fish pathogens [39], similar to the antibacterial spectrum reported for LAB1, LAB10, and LAB11. Using Bakta and DFAST, we identified a conserved gene cluster for bacteriocin production and regulation in all three genomes, going beyond the single *uviB* gene. This cluster encodes two immunity proteins, a bacteriocin, and a pheromone precursor for quorum-sensing control typical of LAB. While functional bacteriocin production was not assayed directly in this study, this expanded gene set is consistent with, and provides additional plausible genomic support for, the broad-spectrum antibacterial phenotype observed against the selected pathogens. Independent, genome-wide corroboration of the probiotic potential was obtained with the ProbML classifier. Across all three clonally identical strains, the per-contig probability scores displayed a clear bimodal distribution. Sixteen contigs, corresponding to the main chromosomal backbone, received scores in the high-probability range (0.80 to 1.00), with 38.2 percent of contigs exceeding 0.90. In contrast, thirteen contigs clustered in the low-probability range (0.20 to 0.40), corresponding to smaller, high-coverage contigs that represent plasmid or mobile genetic element sequences. This separation confirms that the chromosomal architecture of LAB1, LAB10, and LAB11 carries a signature characteristic of recognised probiotic organisms, while mobile elements bearing resistance determinants were appropriately distinguished by the classifier.

The genomic basis for PGP activities is both specific and broad, consistent with recent discoveries that continue to uncover non-canonical genomic pathways responsible for plant-beneficial traits, from alternative auxin biosynthesis routes to novel mechanisms of nutrient mobilisation. Despite the confirmed production of indole-3-acetic acid (IAA) by LAB11, no canonical IAA biosynthesis genes (ipdC, tso) were identified in any of the three genomes. The trpS and miaA genes present in all three assemblies are non-specific housekeeping genes required for tryptophanyl-tRNA charging and tRNA modification in all bacteria; they indicate that the tryptophan/tRNA machinery is intact but are not specific markers of, nor evidence for, any particular IAA biosynthetic route. Non-canonical degradation routes yielding IAA from tryptophan have been documented in several other LAB, but resolving the precise pathway operating in LAB11 will require targeted metabolomics or enzyme activity assays, which were beyond the scope of the present genomic study. Separately, and with the caveat noted regarding the variance in the control group, the observed mean enhancement of maize germination (+23 percentage points) and radicle length (+2.06 mm) by LAB11 cell-free supernatant is directionally consistent with reports for other LAB-derived IAA producers. For example, similar biostimulant effects on root elongation and seed germination have been reported for *E. faecium* on cherry and *L. plantarum* on tomato [27]. Phosphate solubilisation was measured as clear halos on NBRIP agar. The four phosphatase genes identified in all three strains (*acyP*, *uppP*, *gph*, *pspA*) encode intracellular enzymes acting on phosphorylated intermediates, and no determinants of mineral phosphate solubilisation from tricalcium phosphate were detected. Halo formation is therefore more plausibly attributable to organic acid-mediated acidification than to enzymatic release. These enzymes facilitate the release of inorganic phosphate from organic complexes, and the GO term “phosphorus metabolic process” (GO:0006793) (acyP, uppP, gph, pspA) was significantly enriched among the 279 gene clusters exclusive to the three LAB isolates. The absence of potassium solubilisation in phenotypic assays aligns with the lack of dedicated gluconic acid dehydrogenase or potassium feldspar dissolution pathways in the genomes. The PGPT prediction performed with the PLaBAse PGPT-Pred module revealed a rich functional range of traits in all three strains, revealing 994 to 995 plant growth-promoting trait associations per genome. The largest category, Colonising Plant System (34.6 to 34.7 percent), is dominated by plant-derived substrate utilisation functions, including an extensive group of carbohydrate transport and metabolism systems, organic acid utilisation pathways, and glycoside hydrolases, all of which support root colonisation and rhizosphere competence. Competitive Exclusion and Stress Control/Biocontrol categories together account for 38.8 percent of the annotations and include CRISPR-Cas adaptive immunity, multiple multidrug efflux pumps, bacteriocin immunity, and a range of oxidative, osmotic, acid, and temperature stress tolerance genes. Bio-Fertilisation (10.5 percent) is predominantly represented by phosphate solubilisation, potassium solubilisation, nitrogen acquisition, and iron acquisition. Phytohormone and Plant Signal Production (8.1 percent) includes pathways for auxin, cytokinin, gibberellin, and gamma-aminobutyric acid (GABA) biosynthesis, as well as the volatile 2,3-butanediol/acetoin pathway, which is known to trigger induced systemic resistance in plants. The biological consistency of these predictions demonstrates that the chromosomal backbone of these strains contains a substantial plant-beneficial potential, even if some pathways are differentially regulated under the assay conditions used.

The net ammonia-oxidising activity of LAB1 and LAB10 in the absence of the canonical autotrophic nitrification genes *amoA*, *amoB*, and *amoC* establishes that these strains contribute to nitrogen cycling through heterotrophic rather than chemoautotrophic mechanisms [40]. Heterotrophic nitrification is now recognised as a quantitatively significant process in aquaculture pond environments; a comprehensive review of nitrogen-cycling microbial communities in Chinese aquaculture ponds concluded that heterotrophic bacteria operate in parallel with autotrophic Nitrosomonas-type nitrifiers and may dominate nitrogen turnover in high-organic-load systems [41]. The nitrate concentrations achieved by LAB1 (110 mg/L) and LAB10 (80 mg/L) in trout pond water are comparable to values reported for cold-resistant heterotrophic ammonium-removing bacteria evaluated under similar in vivo aquaculture conditions, where *Pseudomonas* and *Acinetobacter* strains lacking canonical *amoA* genes improved water quality metrics in rainbow trout systems [42]. One candidate catabolic route involves the *eutB/eutC*-encoded ethanolamine ammonia-lyase, a vitamin B12-dependent enzyme that cleaves ethanolamine into acetaldehyde and free ammonia; we emphasise that this route is inferred from gene presence alone and has not been experimentally verified in these strains. Under pond water incubation conditions, this liberated ammonia might be directly assimilated by the *gdhA*-encoded glutamate dehydrogenase or released into the medium, where it could support the resident autotrophic nitrifying community and potentially contribute to the accumulation of nitrate observed in assay supernatants; enzymatic assays or gene-knockout studies would be required before this pathway can be considered demonstrated rather than plausible. The most plausible catabolic route involves the *eutB/eutC*-encoded ethanolamine ammonia-lyase, a vitamin B12-dependent enzyme that cleaves ethanolamine, a nitrogen-containing membrane phospholipid breakdown product, into acetaldehyde and free ammonia. Under pond water incubation conditions, this liberated ammonia may be directly assimilated by the *gdhA*-encoded glutamate dehydrogenase or released into the medium, where it may support the resident autotrophic nitrifying community and account for the accumulation of nitrate observed in assay supernatants.

The strain-differential nitrifying phenotype observed between LAB10 (highest in vitro nitrate) and LAB1 (highest in vivo nitrate) cannot be explained by gene content alone, since all three assemblies encode an identical *eutBC/gdhA* complement. Differences in enzyme expression levels, regulatory responses to the aquatic matrix, or competitive dynamics with the resident pond microbiota would more likely explain our observation. However, transcriptomic experiments under nitrogen-replete and nitrogen-limited conditions, paired with enzyme activity assays, would be required to resolve the mechanistic basis of the observed strain-level variation in nitrifying capacity.

## 5. Conclusions

In summary, the integrated genomic evidence positions LAB1, LAB10, and LAB11 as clonal, multifunctional *E. faecalis* strains with a chromosomal backbone consistent with probiotic and plant growth-promoting activities. The safety profile is favourable in terms of absent cytolysin and vancomycin resistance, but the plasmid-borne *tet(M)* gene requires risk mitigation, such as plasmid curing or demonstration of non-transferability, prior to any applied use.

## Figures and Tables

**Table 2:** Virulence factor homologs detected by VFDB screening in all three *Enterococcus faecalis* strains.

Gene	Virulence Category	Product	% Identity	% Coverage
*cpsA*	Capsule (VF0361)	Undecaprenyl diphosphate synthase	99.14	100.0
*cpsB*	Capsule (VF0361)	Phosphatidate cytidylyltransferase	98.75	100.0
*EF0818*	Hyaluronidase (VF0359)	Polysaccharide lyase family 8	99.57	100.0
*fsrB*	Fsr QS system (VF0360)	AgrBfs accessory protein	98.63	100.0
*fsrC*	Fsr QS system (VF0360)	Histidine kinase (putative)	98.81	100.0
*gelE*	Gelatinase (VF0357)	Coccolysin (GelE metalloprotease)	98.96	100.0
*sprE*	Serine protease (VF0358)	Serine proteinase V8 family	98.48	100.0
*ebpA*	Ebp pili (VF0538)	Pilus tip adhesin EbpA	99.94	100.0
*ebpB*	Ebp pili (VF0538)	Pilus minor subunit EbpB	99.16	100.0
*ebpC*	Ebp pili (VF0538)	Pilus major subunit EbpC	99.05	100.0
*srtC*	Ebp pili (VF0538)	Sortase C (pilus assembly)	99.30	100.0
*efaA*	EfaA (VF0354)	Endocarditis-specific antigen EfaA	99.57	100.0
*bopD*	BopD (VF0362)	LacI-family transcriptional regulator	99.11	100.0
*fss1*	Fibrinogen binding (AI271)	Surface protein Fss1 (fibrinogen-binding)	98.62	100.0

**Table 3:** Selected functional genes detected by Prokka annotation in all three *Enterococcus faecalis* strains.

Category	Gene	Product	LAB1	LAB10	LAB11
Stress resistance	*katA*	Vegetative catalase	+	+	+
Stress resistance	*ydbD*	Putative manganese catalase	+	+	+
Stress resistance	*sodA*	Superoxide dismutase [Mn]	+	+	+
GI tolerance	*cbh*	Choloylglycine hydrolase (BSH)	+	+	+
Antimicrobial	*uviB*	Bacteriocin UviB	+	+	+
Antimicrobial/Virulence	*gelE*	Gelatinase (coccolysin)	+	+	+
Siderophore transport	*fepC*	Ferric enterobactin transporter (ABC-type)	+	+	+
Siderophore transport	*fhuD*	Iron(III)-hydroxamate binding protein	+	+	+
N-metabolism	*eutB*	Ethanolamine ammonia-lyase heavy chain	+	+	+
N-metabolism	*eutC*	Ethanolamine ammonia-lyase light chain	+	+	+
N-metabolism	*dpaL*	Diaminopropionate ammonia-lyase	+	+	+
IAA-related	*trpS*	Tryptophan–tRNA ligase	+	+	+
IAA-related	*miaA*	tRNA dimethylallyltransferase	+	+	+
Safety: ARG	*tet(M)*	Tetracycline resistance (ribosomal protection)	+	+	+
Safety: ARG	*lsa(A)*	Lincomycin/Clindamycin ABC-F resistance	+	+	+
Safety: absent	*vanA/B/C*	Vancomycin resistance	−	−	−
Safety: absent	*cylA–M*	Cytolysin operon	−	−	−
Heat shock/Stress tolerance	*groEL*	GroEL chaperonin, large subunit (Hsp60)	+	+	+
Heat shock/Stress tolerance	*groES*	GroES chaperonin co-chaperone (Hsp10)	+	+	+
Heat shock/Stress tolerance	*dnaK*	DnaK heat shock protein (Hsp70)	+	+	+
Adhesion/Colonisation	*ebpA*	Endocarditis- and biofilm-promoting pilus tip adhesin EbpA	+	+	+
Adhesion/Colonisation	*ebpB*	Pilus minor subunit EbpB	+	+	+
Adhesion/Colonisation	*ebpC*	Pilus major subunit EbpC	+	+	+
Adhesion/Colonisation	*srtC*	Sortase C (pilus assembly)	+	+	+
Adhesion/Colonisation	*efaA*	Endocarditis-specific antigen EfaA (surface adhesin)	+	+	+
Adhesion/Colonisation	*fss1*	Surface protein Fss1 (fibrinogen-binding)	+	+	+
IAA-related	*trpS2*	Tryptophan–tRNA ligase II (alternate tryptophan activation)	+	+	+
Phosphate solubilisation	*acyP*	Acylphosphatase	+	+	+
Phosphate solubilisation	*uppP*	Undecaprenyl pyrophosphate phosphatase	+	+	+
Phosphate solubilisation	*gph*	Phosphoglycolate phosphatase	+	+	+
Phosphate solubilisation	*pspA*	Phage shock protein A (phosphatase activity)	+	+	+
N-metabolism	*gdhA*	Glutamate dehydrogenase (oxidative deamination/N-assimilation)	+	+	+

## Data Availability

The draft genome sequences of LAB1, LAB10, and LAB11 have been deposited in the NCBI GenBank database under BioProject PRJNA1453999. The BioSample accessions are SAMN57287360 (LAB1), SAMN57287361 (LAB10), and SAMN57287362 (LAB11). The Whole Genome Shotgun (WGS) project accession numbers are JBXPLQ000000000 (LAB1), JBXPLP000000000 (LAB10), and JBXPLO000000000 (LAB11). The 16S rRNA gene sequences are deposited under GenBank accessions PX518070 (LAB1), PX518071 (LAB10), and PX518072 (LAB11). Raw sequencing reads are available through the same BioProject (PRJNA1453999) via the NCBI Sequence Read Archive. A detailed summary of the phenotypic screening, genome annotation, AMR genes, and virulence genes has been made available on Figshare (https://doi.org/10.6084/m9.figshare.32524470).

## Supplementary Materials

The following supporting information can be downloaded at: https://www.mdpi.com/article/10.3390/microorganisms14081653/s1, Table S1. Representative probiotic probability scores computed per contig for LAB1. Scores represent the probability assigned to each contig of belonging to a probiotic genome vs. a non-probiotic ge-nome, computed by ProbML. Contigs ≥ 20 kbp (n = 15 main chromosomal contigs, NODE 1–15) predominantly score > 0.87. Bold values indicate probiotic probability ≥ 0.87. Coverage values for NODE 27–34 exceed chromosomal mean indicative of extrachromosomal or plasmid-derived elements.
