# Supplementary material for: Genome Sequences of Three Enterococcus faecalis Strains (LAB1, LAB10, and LAB11) with Probiotic, Plant Growth-Promoting, and Nitrifying Properties
Source: Microorganisms. 2026 Jul 29;14(8):1653. doi: 10.3390/microorganisms14081653 (PMC13515994; doi:10.3390/microorganisms14081653)
Supplement: Supplementary file 1 [file microorganisms-14-01653-s001.zip › microorganisms-4400136-supplementary.pdf]

Genome Sequences of Three *Enterococcus faecalis* Strains (LAB1, LAB10, and LAB11) with Probiotic, Plant Growth-Promoting, and Nitrifying Properties

Wahauwouélé Hermann Coulibaly[1], Muiz O. Akinyemi [2,3], Tano Marie-Ange Sakia Mian[4], Paul-Alexandru Popescu[5], Thierry Fievez[6], Phillipe Maesen[6], Hary Razafindralambo[7,8,9,10,*]

[1]Biotechnology and Food Microbiology Laboratory Food Science and Technology Formation and Research Unit, Nangui Abrogoua University, 02 PO Box 801 Abidjan 02, Côte d'Ivoire

[2]Leeds Institute of Health Sciences, University of Leeds, Leeds, LS2 9LN, UK

[3]Unit for Environmental Sciences and Management, North-West University, Potchefstroom, South

[4]Agro-Valorization Laboratory, Agroforestery Trainning and Research Unit, Jean Lorougnon Guédé University, Daloa, BP-150, Ivory Coast

[5]University of Agronomic Sciences and Veterinary Medicine of Bucharest,59 Marasti Blvd, District 1, Bucharest, Romania

[6]BEAGx, Gembloux Agro-Bio Tech, University of Liege, 2 Passage Des Déportés, B-5030, Gembloux, Belgium

[7]Microbial Processes and Interactions, TERRA Teaching and Research Centre, UMRt 1158 BioEcoAgro, Gembloux Agro-Bio Tech, University of Liege, Gembloux, Belgium

[8]ProBioLab, 5004 Namur, Belgium

[9]An Giang University & Vietnam National University Hochiminh, Thu Duc, 71357, Ho Chi Minh city, Vietnam 18 Ung Van Khiem street, Long Xuyen, An Giang, 90100, Vietnam

[10]Université de Vakinankaratra, Ingénierie et Technologie Emergentes, Campus Vatofotsy Antsirabe 110 BP 108, Madagascar

*Corresponding author: h.razafindralkambo@uliege.be

ORCID: 0000-0002-4757-7072

Table S1. Representative probiotic probability scores computed per contig for LAB1. Scores represent the probability assigned to each contig of belonging to a probiotic genome vs. a non-probiotic genome, computed by ProbML. Contigs ≥20 kbp (n = 15 main chromosomal contigs, NODE 1-15) predominantly score >0.87. Bold values indicate probiotic probability ≥0.87. Coverage values for NODE 27–34 exceed chromosomal mean indicative of extrachromosomal or plasmid-derived elements.

Contig	Length (bp)	Coverage (×)	Probiotic Probability	Non-Probiotic Probability
**Contigs ≥ 20 kbp (n = 20; chromosomal backbone NODE_1–NODE_15 shaded blue)**				
NODE_1	376,685	237.09	**0.9647**	0.0353
NODE_2	369,923	187.89	0.7294	0.2706
NODE_3	272,898	201.63	**0.9896**	0.0104
NODE_4	260,287	283.39	**0.9983**	0.0017
NODE_5	248,560	208.84	**0.8742**	0.1258
NODE_6	213,359	217.25	**0.9890**	0.0110
NODE_7	156,946	264.15	**0.9650**	0.0350
NODE_8	156,487	282.95	**0.9702**	0.0298
NODE_9	144,012	241.31	**0.9134**	0.0866
NODE_10	128,010	277.64	**0.9377**	0.0623
NODE_11	107,412	230.34	**0.9936**	0.0064
NODE_12	84,866	194.21	0.8388	0.1612
NODE_13	79,956	266.60	**0.9689**	0.0311
NODE_14	69,651	196.55	**0.9429**	0.0571
NODE_15	62,845	249.41	**0.9889**	0.0111
NODE_16	27,962	266.68	0.3617	0.6383
NODE_17	27,686	234.11	**0.9111**	0.0889
NODE_18	27,438	207.26	0.2481	0.7519
NODE_19	25,907	248.00	0.4526	0.5474
NODE_20	20,507	226.28	0.6047	0.3953
**Contigs < 20 kbp**				
NODE_21	16,855	186.74	0.4336	0.5664
NODE_22	15,504	218.86	0.8419	0.1581
NODE_23	15,207	251.12	0.1899	0.8101
NODE_24	8,784	247.55	0.2063	0.7937
NODE_25	5,694	205.40	0.2188	0.7813
NODE_26	5,489	197.27	0.2520	0.7480

NODE_27	4,901	3595.44	0.2360	0.7640
NODE_28	3,382	1076.10	0.2459	0.7541
NODE_29	1,777	693.77	0.2456	0.7544
NODE_30	1,517	929.03	0.2456	0.7544
NODE_31	1,415	281.97	0.2456	0.7544
NODE_32	1,066	1117.03	0.2456	0.7544
NODE_33	579	0.73	0.2456	0.7544
NODE_34	578	1143.57	0.2456	0.7544

Abbreviations: bp, base pairs; ×, times coverage; Probiotic Probability, XGBoost classifier score for probiotic classification; Non-Probiotic Probability = 1 − Probiotic Probability.
